# Genome‐wide association study of an unusual dolphin mortality event reveals candidate genes for susceptibility and resistance to cetacean morbillivirus

**DOI:** 10.1111/eva.12747

**Published:** 2018-12-26

**Authors:** Kimberley C. Batley, Jonathan Sandoval‐Castillo, Catherine M. Kemper, Catherine R. M. Attard, Nikki Zanardo, Ikuko Tomo, Luciano B. Beheregaray, Luciana M. Möller

**Affiliations:** ^1^ Molecular Ecology Laboratory, College of Science and Engineering Flinders University Adelaide South Australia Australia; ^2^ Cetacean Ecology, Behaviour, and Evolution Laboratory, College of Science and Engineering Flinders University Adelaide South Australia Australia; ^3^ South Australian Museum Adelaide South Australia Australia

**Keywords:** bottlenose dolphin, cetacean morbillivirus, ecological genomics, genome-wide association study, immune genes, pathogen resistance, virus, wildlife disease

## Abstract

Infectious diseases are significant demographic and evolutionary drivers of populations, but studies about the genetic basis of disease resistance and susceptibility are scarce in wildlife populations. Cetacean morbillivirus (CeMV) is a highly contagious disease that is increasing in both geographic distribution and incidence, causing unusual mortality events (UME) and killing tens of thousands of individuals across multiple cetacean species worldwide since the late 1980s. The largest CeMV outbreak in the Southern Hemisphere reported to date occurred in Australia in 2013, where it was a major factor in a UME, killing mainly young Indo‐Pacific bottlenose dolphins (*Tursiops aduncus*). Using *cases* (nonsurvivors) and *controls* (putative survivors) from the most affected population, we carried out a genome‐wide association study to identify candidate genes for resistance and susceptibility to CeMV. The genomic data set consisted of 278,147,988 sequence reads and 35,493 high‐quality SNPs genotyped across 38 individuals. Association analyses found highly significant differences in allele and genotype frequencies among *cases* and *controls* at 65 SNPs, and Random Forests conservatively identified eight as candidates. Annotation of these SNPs identified five candidate genes (*MAPK8*, *FBXW11*, *INADL*, *ANK3* and *ACOX3*) with functions associated with stress, pain and immune responses. Our findings provide the first insights into the genetic basis of host defence to this highly contagious disease, enabling the development of an applied evolutionary framework to monitor CeMV resistance across cetacean species. Biomarkers could now be established to assess potential risk factors associated with these genes in other CeMV‐affected cetacean populations and species. These results could also possibly aid in the advancement of vaccines against morbilliviruses.

## 1 INTRODUCTION

Infectious diseases are caused by pathogens such as viruses, bacteria and parasites and are significant demographic and evolutionary drivers of human, domestic and wildlife populations (Acevedo‐Whitehouse & Cunningham, 2006). Novel pathogens are emerging, others re‐emerging and some becoming resistant to antimicrobial agents (Cunningham, Daszak, & Wood, 2017; Daszak, Cunningham, & Hyatt, 2000; di Guardo, Mazzariol, & Fernández, 2011; Karlsson, Kwiatkowski, & Sabeti, 2014; Van Bressem et al., 2009). Posing a threat to individuals and populations, infectious diseases can trigger large morbidity and mortality events, induce loss of genetic diversity and lead to population declines and extinction (Roelke‐Perker et al., 1996; Thorne & Williams, 1998; Van Bressem et al., 2014). Host genetic factors associated with the innate and adaptive immune systems are well known to be major determinants of susceptibility and resistance to infections in humans and vertebrate model organisms (Hill, 2001; Karlsson et al., 2014; Martin & Carrington, 2005), but studies of the genetic basis of host defence against infectious diseases in wildlife populations are still scarce (Loiseau et al., 2011; Queiros, Vicente, Alves, de la Fuente, & Gortazar, 2016; Wright et al., 2017). Investigating how variants in genes associated with infectious diseases are generated and maintained in wildlife hosts is key to understanding the genetic basis of their immune system, and the prospects for individual survival and population persistence (Acevedo‐Whitehouse & Cunningham, 2006).

Among viruses, morbilliviruses are highly contagious and virulent diseases that belong to the family of RNA viruses Paramyxoviridae, which show frequent cross‐species transmissions (Geoghegan, Duchene, & Holmes, 2017). This family contains seven genera, including Morbillivirus, which includes seven viral species that infect mammals. These include the measles virus (MV) in humans, phocine distemper virus (PDV) in true seals, rinderpest in cattle, canine distemper virus (CDV) in carnivores, feline morbillivirus in felids and peste des petits ruminants virus in ruminants (Alfonso et al., 2016). The seventh viral species is the cetacean morbillivirus (CeMV), which includes three described strains: dolphin, porpoise and pilot whale morbilliviruses (Sacristán et al., 2015). All seven viral species are distinct, but share similar structure, genome and pathological characteristics (da Fontoura Budaszewski & von Messling, 2016; Kennedy et al., 1991). Knowledge of disease pathology and immune responses may therefore relate to morbillivirus species in general (da Fontoura Budaszewski & von Messling, 2016). In the case of MV, the immune response to its main structural proteins is thought to be controlled by T lymphocytes and *HLA* class *I* (B) and class *II* *(DQA, DQB, DRB) *molecules, but polymorphisms in several other genes also seem to play a role in defence: binding genes, *SLAM* and *CD46*; pathogen‐associated molecular patterns sensing genes, *TLRs*, *CD209*; cytokine/cytokine receptor genes, *IL2, IL10, TNFA*; antiviral genes, *TRIM5, ADAR, MX2*; vitamin A and D receptor genes, *RARA, RARB*, among others (Haralambieva, Kennedy, Ovsyannikova, Whitaker, & Poland, 2015). Several of these genes are also believed to be involved in defence against morbillivirus infections in other host species (Hashiguchi et al., 2011; Melia et al., 2014; Woodman et al., 2016).

Morbilliviruses are extremely infectious (Diallo, 1990), with CeMV outbreaks likely to cause serious harm and possibly death in most of the immunologically naïve individuals of a population (Van Bressem et al., 2014). In humans, MV immunity in at least 94% of a population is required to interrupt endemic transmission of this virus (Boulton et al., 2016), but there is still large interindividual variation in immune response to the measles vaccine (Haralambieva et al., 2015). Morbilliviruses are generally highly fatal, with a mortality rate of about 70%–80% (Diallo et al., 2007), and typically have a high rate of transmission, with the potential for one infected individual to transmit the virus to approximately 15–20 others (Griffin, Pan, & Moss, 2008). In the case of CeMV, it can be transferred throughout a cetacean population via the inhalation of aerosolized virus particles during synchronous breathing (Morris et al., 2015), or by the direct transmission of bodily fluids (Van Bressem et al., 2014), which can be exacerbated by the highly social nature and promiscuity of many cetacean species (Möller, 2012). CeMV‐infected cetaceans can suffer from fatal acute systemic disease affecting the lymphoid system, develop bronchopneumonia or nonsuppurative encephalitis and can also develop serious secondary systemic infections from bacteria, fungi and parasites (Di Guardo & Mazzariol, 2016).

**Figure 1:**
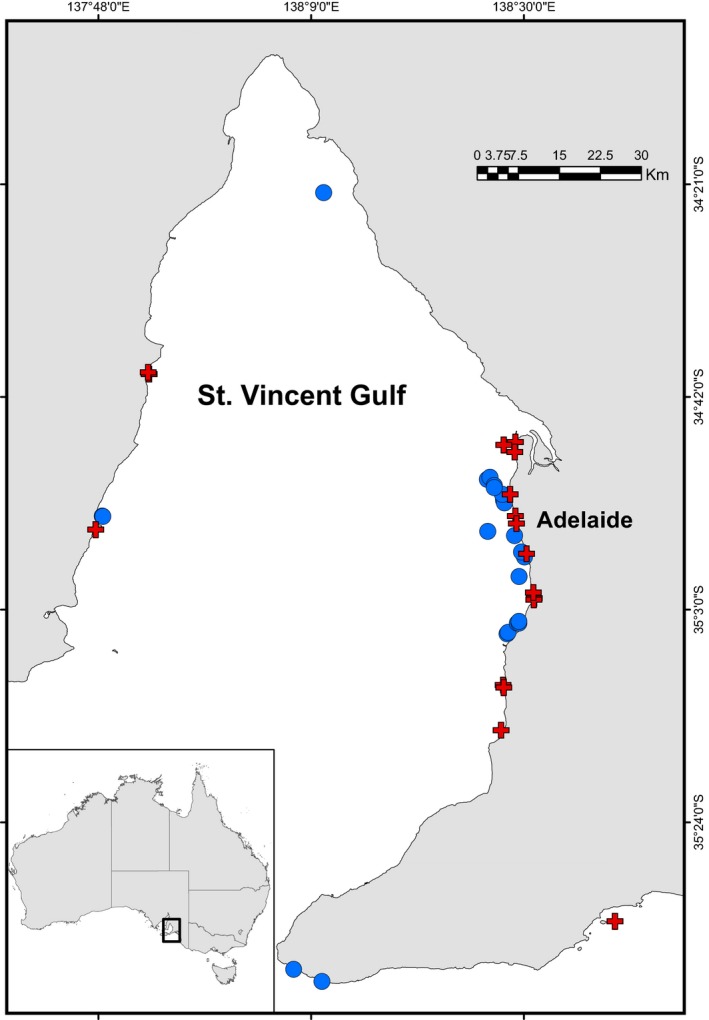
Locations of Indo‐Pacific bottlenose dolphin (*Tursiops aduncus*) *case* (*n = *17) (cross) and *control* (*n = *21) (circle) samples used for a genome194 10 wide association study of cetacean morbillivirus susceptibility and resistance in the St. Vincent Gulf bioregion, South Australia. The map was created using ArcGIS 10.4.1 (Esri) with coastline boundaries from DIVA‐GIS (http://http.diva-gis.org/Data)

Recent increase in the reporting of disease outbreaks and unusual mortality events (UME) of cetacean populations has raised concerns over the seemingly declining health of populations and their ecosystems (Gulland & Hall, 2007). The term “UME” relates to the unexpected deaths within a cetacean population at densities greater than the annual mean number of deaths, which demands a rapid response by managers (Kemper et al., 2016). In cetaceans, UMEs have been caused by pathogens, harmful algal blooms (HAB’s), bacteria and parasites, exposure to pollutants and nutrients, changes in oceanographic conditions, or human interactions (Cammen, Schultz, Rosel, Wells, & Read, 2015; Gulland & Hall, 2007; Kemper et al., 2016). Of these, CeMV has emerged as a significant threat since the late 1980s (Van Bressem et al., 2014), leading to UMEs of dolphins worldwide, particularly in the Northern Hemisphere, resulting in the death of tens of thousands of individuals (Morris et al., 2015; Raga et al., 2008). In the Southern Hemisphere, a UME and the largest recorded CeMV outbreak so far occurred between March and September of 2013 in South Australia (SA), which led to the stranding and death of at least 50 dolphins from three different species (short‐beaked common dolphins, *Delphinus delphis*; common bottlenose dolphins, *Tursiops truncatus*; and Indo‐Pacific bottlenose dolphins; *Tursiops aduncus*; Kemper et al., 2016). While the cause for the UME appears to be multifactorial, evidence of CeMV infection was found in dead individuals of all three species, and all bottlenose dolphins tested positive for CeMV (Kemper et al., 2016). The majority of the deaths, however, were represented by Indo‐Pacific bottlenose dolphins from the St. Vincent Gulf bioregion (SVG; Figure 1; Kemper et al., 2016). These dolphins are known to belong to one genetic population, which show a relatively small abundance (approximately 700–1,200 dolphins), high social connectivity and low genetic diversity (Pratt et al., 2018; Zanardo, Parra, & Möller, 2016). These factors, accompanied by a highly transmissible virus, have the potential to lead to a population decline.

The role of the immune system in combatting CeMV is understudied (Van Bressem et al., 2014), and this is especially true for the genetic component of the host–infection interaction. However, understanding the role that genetic variation plays in susceptibility and resistance of individuals to this infectious disease is vital for identifying populations at risk and predicting their evolutionary potential. Advancements in next‐generation sequencing techniques, and in particular the development of RADseq approaches (Baird et al., 2008; Peterson, Weber, Kay, Fisher, & Hoekstra, 2012), have enabled the move from traditional genetic studies that are limited to prior knowledge of candidate genes, or tens of markers (Attard, Beheregaray, Sandoval‐Castillo, et al., 2018; Cammen, Schultz, et al., 2015), to large data sets well suited to address the genetic basis of adaptation in wildlife populations (Catchen et al., 2017; Peterson et al., 2012).

Here, we capitalize on the opportunity provided by the 2013 SA UME to generate ddRAD (double digest restriction site‐associated DNA) data and implement a genome‐wide association study (GWAS) to investigate the genetic basis of resistance and susceptibility to CeMV, using *case* (nonsurvivors) and *control* (putative survivors) Indo‐Pacific bottlenose dolphins. To the best of our knowledge, this study provides the first empirical evidence for candidate genes associated with CeMV immunity. Our results can be used for developing biomarkers for these genes, which can be applied for screening cetacean populations worldwide, and possibly other mammalian groups due to the cross‐species capabilities of morbilliviruses. This may lead to the identification of other potentially susceptible populations and possibly promote future conservation and management strategies to minimize the risk and severity of outbreaks.

## 2 MATERIALS AND METHODS

### 2.1 Sample collection

Samples were collected from a single and small genetic population of *T. aduncus* that resides in the SVG bioregion (Pratt et al., 2018) and includes individuals that are *case* (nonsurvivors) and *control* (putative survivors) of the 2013 SA UME and CeMV outbreak (Figure 1).

Samples from *cases* that died during the UME were collected by the South Australian Museum (SAM) between March and September 2013. Histopathological examinations, reverse‐transcription polymerase chain reaction (RT‐PCR) and/or immunohistochemical assays (IHC) confirmed that most of the dolphins died from CeMV infection and related pathologies (Kemper et al., 2016). From the 29 samples available at SAM from the SVG bioregion that died during the UME, a subset of 26 dolphins (neonates and calves, *n* = 13; juveniles, *n* = 10; adults, *n* = 3; neonates, calves and juveniles *ca.* <1.6 m and adults >1.6 m) that tested positive for RT‐PCR and/or IHC were selected as *cases*. Their general pathology was interstitial pneumonia, lymphoid depletion and syncytia (Kemper et al., 2016).

Biopsy samples from 153 free‐ranging juvenile and adult dolphins from the same small genetic population were collected during 2014 and 2015 (Zanardo et al., 2016), within 18 months after the end of the outbreak. They were collected using either the PAXARMS system (Krützen et al., 2002) or a hand‐held biopsy pole (Bilgmann, Möller, Harcourt, Gibbs, & Beheregaray, 2007). The age class (juvenile, adult) of *controls* was estimated in the field and/or photographically based on body size and degree of independence from an adult dolphin (Zanardo et al., 2016). Samples from neonates and calves were not collected due to conditions in the research permit. Biopsy samples were preserved in a salt‐saturated solution of 20% DMSO and stored at −80°C. Dolphins were genetically sexed using the method described in Banks, Levine, Syvanen, Theis, and Gilson (1995). From the available samples, 24 dolphins were selected (juvenile, *n* = 14; adult, *n* = 10), along with two stranded juveniles that died from confirmed causes unrelated to CeMV (sampled by the SAM in 2015), to represent *controls*. Since most dolphins that died from CeMV infection during the UME were young animals (neonates, calves and juveniles), juveniles were preferentially chosen for the *control* group to minimize the influence of age structure in our *case *and *control *comparison. These *control* juveniles likely represent individuals that were calves during the UME. This resulted in all samples available from juveniles being selected for the *control *group, together with a random sample of adults.

### 2.2 Data collection

#### 2.2.1 Laboratory methods

Genomic DNA was extracted using the salting out method (Sunnucks & Hales, 1996). The quality of DNA extractions was verified using a spectrophotometer (NanoDrop, Thermo Scientific), quantity using a fluorometer (Qubit, Life Technologies), and integrity using 2% agarose gels. Extractions that failed initial quality control were re‐extracted, and those showing degradation after re‐extraction were size‐selected for high molecular weight DNA using magnetic beads (Agencourt AMPure XP). Of the original 26 *cases* and 26 *controls *obtained for DNA extractions, only samples that were of high enough quantity and quality for genomic library preparation (17 *cases* and 22 *controls*), together with a single replicate from each group, were used for library preparation (Supporting Information Table S1).

Libraries were prepared following the ddRAD protocol of Peterson et al. (2012), with modifications as described in Brauer, Hammer, and Beheregaray (2016). In summary, 300 ng of genomic DNA per sample was digested using the restriction enzymes *SbfI* and *MseI*, with a unique 6 bp barcode adaptor ligated to each individual library. *Cases *and *controls* were multiplexed into a final pool of 41 samples with equal molar concentrations, and fragments between 250 and 800 bp were selected using a Pippin Prep (Sage Science) machine. The resulting library was paired‐end 125 bp sequenced on one lane of Illumina HiSeq2500 at the Genome Quebec Innovation Centre of McGill University.

#### 2.2.2 Bioinformatics

The resulting reads were demultiplexed using the “process_radtags.pl” program in STACKS 1.19 (Catchen, Hohenlohe, Bassham, Amores, & Cresko, 2013), allowing a maximum of two mismatches in the barcode (barcodes are unique up to two mismatches) and RAD tags (restriction enzyme recognition sequence). Remaining reads were trimmed to 113 bp (forward) and 122 bp (reverse) by removing barcodes and RAD tags. The dDocent 2.18 pipeline (Puritz, Hollenbeck, & Gold, 2014) was used to substitute low‐quality bases (phred score <20) with N’s, with reads containing more than five consecutive N’s eliminated. Within the dDocent pipeline, sequencing reads were aligned de novo using a minimum coverage of 15×, and a maximum of 12 mismatches were allowed to form reference contigs. After this, the sequencing reads of each sample were mapped to the reference contigs with an 80% similarity threshold, creating alignment files for each sample for each contig.

From the aligned reads of all individuals, SNPs were called and first filtered using the dDocent pipeline, which combines pre‐existing software packages into one pipeline (Brauer et al., 2016; Puritz et al., 2014). Specifically, SNPs were called in FREEBAYES (Garrison & Marth, 2012) and first filtered using vcftools (Danecek et al., 2011) as described in Brauer et al. (2016), with the following modifications: SNPs with a minor allele frequency <5%, genotyped in <80% of the samples, and with allele balance <0.2 and >0.8 discarded (calculated per locus across heterozygous sites). In addition, replicate samples were compared to ensure that genotyping error was <5%. The replicate sample with the highest amount of missing data was removed from further analysis. The resulting vcf file was converted into the PLINK format. The SNP data set was further filtered using GenABEL 1.8‐0 (Aulchenko, Ripke, Isaacs, & van Dujin, 2007) in R 3.1.0 (R. C. Team, 2014) within the RStudio 0.98.1102 environment (R. Team, 2015) to remove SNPs and individuals with a low call rate (<95%). SNPs out of Hardy–Weinberg equilibrium (HWE) were also excluded (false discovery rate <0.2) as these can be indicative of genotyping errors (Laurie et al., 2010), as well as individuals with high identity‐by‐state (>0.95) values, which would be suggestive of unintended duplicate *controls*, which could result from duplicate biopsy sampling of the same individual**.** The quality‐filtered reads were mapped to the *T. truncatus* genome (Tur_tru v1, GenBank Assembly ID: GCF_001922835.1; Lindblad‐Toh et al., 2011) using Bowtie2 (Langmead & Salzberg, 2012) with standard parameters, allowing no mismatches in seed alignment and up to 20 consecutive seed fails (Table 1 and Supporting Information Table S2 for resulting depth per contig per individual).

**Table 1:** Number of SNPs retained after each filtering step using dDocent and GenABEL, for the Indo‐Pacific bottlenose dolphin (*Tursiops aduncus*)

Step	SNP count
dDocent filtering	
Raw SNP catalogue	215,067
Genotyped in 80% of individuals, base quality ≥30, minor allele frequency>0.05 and bi‐allelic	77,920
Sequencing errors, paralogs, multicopy loci and artefacts of library preparation	
(1) Allele balance (>20% and <80%)	71,487
(2) Mapping alleles quality ratio (>0.9 and <1.05)	60,815
(3) Read quality (ratio quality/coverage depth >0.2)	60,534
(4) Read depth (≤mean depth + [2* standard deviation])	59,191
(5) Removed indels	55,365
(6) Present in 90% of individuals	51,499
(7) 95% of SNPs present in an individual (1 individual removed)	48,882
GenABEL filtering	
SNPs with low call rate (<95%) and out of HWE (FDR <0.2)	39,082
Mapping to the *Tursiops truncatus* genome	35,493
Outlier detection	
GenABEL outliers (*p* < 0.001)	65
RandomForest outliers	8

### 2.3 Data analyses

#### 2.3.1 Relatedness and inbreeding

Relatedness between individuals was estimated to assess whether there was a potential bias in the association analyses due to significant differences in relatedness between the *cases* and *controls*. Inbreeding coefficients were also estimated to assess whether inbreeding levels potentially increased the risk of dolphins succumbing to CeMV. As COANCESTRY requires unlinked SNPs for these analyses, we estimated the squared correlation coefficient among pairs of contigs using the–geno‐*r* ^2^ function in Vcftools. One contig from each pair with an *R* ^2^ > 0.8 was subsequently removed. Pairwise relatedness between samples and individual inbreeding coefficients was estimated using the Ritland (1996) relatedness and inbreeding estimators within COANCESTRY 1.0.1.8 (Wang, 2011). The Ritland relatedness estimator was chosen as it has shown to perform better than other estimators when using large SNP data sets for cetaceans (Attard, Beheregaray, & Moller, 2018). Differences in mean relatedness and mean inbreeding between *cases* and *controls* (for relatedness, *case–control* values were also estimated) were tested in COANCESTRY using 10^6^ bootstraps.

#### 2.3.2 Genome‐wide association analyses and Random Forests

GenABEL was used to perform association tests to identify SNPs potentially associated with dolphin susceptibility and resistance to CeMV. We used treatment, *case* or *control*, and age class, adult or young, as phenotypic information. The young class included neonates, calves and juveniles for the *cases* and juveniles (at time of sampling) for the *controls*. Chi‐square distributions of alleles and genotypes at each SNP between *cases* and *controls*, corrected for inflation, were used to test for potential associations using the ccfast function in GenABEL. Since the proportion of young to adult individuals (Supporting Information Table S1) was significantly different between *cases* and *controls* (*z*‐score test, *Z* = 2.56, *p* < 0.05), age was also included as an explanatory variable using a basic generalized linear model (GLM), in GenABEL. Sex ratios were similar between *cases* and *controls* (Supporting Information Table S1), with no significant difference in their proportions (*z*‐score test, *Z* *=* −1.28, *p* = 0.20), and therefore, sex was not included as an explanatory variable. SNPs with a highly significant *p*‐value (*p* < 0.001) for each test were selected for subsequent Random Forest (RF) analyses.

Random Forest is a tree‐based ensemble machine‐learning tool, which is highly data adaptive, making it very useful for analysing genomic data (Chen & Ishwaran, 2012). This algorithm is particularly suited for detecting (with a high prediction accuracy) contigs that best explain variation in a response variable (Brieuc, Waters, Drinan, & Naish, 2018), and therefore loci under selection, for data sets with many thousands of SNPs and a relatively small number of samples (Chen & Ishwaran, 2012). RF was used here for identifying SNPs that are putatively under selection and therefore represent candidate SNPs for CeMV‐associated immunity. The randomForest package (Liam & Wiener, 2002) within R was run on the highly significant SNPs from the chi‐square test and the GLM of both allele and genotype frequencies separately (i.e., four independent runs with specific SNP subsets). The RF algorithm does not accept missing data; therefore, we used the na.roughfix function in the randomForest R package to impute missing genotypes (0.17% of missing data). To avoid type I errors due to imputations, we drew alleles from the allele frequency of the entire data set, rather than from each treatment separately. A random two thirds of the samples were used as a training data set to generate the RF, with the remaining samples used to calculate the out‐of‐bag (OOB) error rate. In each RF, 125,000 trees were generated; with between two and six randomly chosen SNPs considered in each tree split (mtry; Supporting Information Table S3). As there is no specific method to select important SNPs using RF (Goldstein, Polley, & Briggs, 2011; Laporte et al., 2016), we selected candidate SNPs using the importance value distributions (mean decrease accuracy, given as the number of times the random trees were inaccurate when permuting that specific SNP, therefore SNP ranking: highest number = greater importance). SNPs above the upper elbow of the distribution curves were selected as candidates. Vote distributions of each sample for each RF analysis were plotted to visualize the classification of each sample. Importance values and classification votes were also permuted (500 times) using the R package rfPermute (Archer, 2016) to obtain *p*‐values for the candidate SNPs of each RF analysis.

Finally, to explore the function of identified candidate SNPs, contigs were aligned against the *T. truncatus* (NIST Tur_tru v1 GenBank Assembly ID: GCF_001922835.1) and the killer whale (*Orcinus orca*) genomes (Oorc_1.1, GenBank Assembly ID: GCA_000331955.1; Pruitt et al., 2014) using Blastn (Altschul, Gish, Miller, Myers, & Lipman, 1990), with candidate genes identified within 20 kb of the candidate contig. To identify the gene regions in which our candidate contigs aligned to, and any putative functional changes, we used the genome data viewer in NCBI (NCBI Resource Coordinators, 2016). Gene functions were then investigated using UniProtKB (Huntley et al., 2015) with an *E*‐value threshold of 1 × 10^−06^.

## 3 RESULTS

### 3.1 SNP data set

A total of 39 *T. aduncus* samples from one genetic population that resides in SVG (Figure 1) were collected between 2013 and 2015 and sequenced using ddRAD. A total of 525,785,802 sequence reads were generated on one lane of the Illumina HiSeq2500 platform (Supporting Information Table S4). After demultiplexing and quality filtering, 278,147,988 reads (mean = 6,784,097 ± *SD* 3,457,688 reads per sample) were obtained (Supporting Information Table S4). De novo assembly and initial SNP filtering within the dDocent pipeline resulted in 55,365 SNPs, and 39,082 SNPs after further quality filtering in GenABEL (Table 1).

The genotypes of replicates were highly similar (99.89% and 99.99%), suggesting that genotype calling was very accurate. Only one *control* sample with low coverage was removed due to a high amount of missing data (12%). No pair of *control *samples with a high identity by state was found, suggesting that all represented unique individuals.

The 39,082 resulting SNPs were located within 22,023 individual contigs. Of these, 19,763 contigs (89.74%) containing 35,493 SNPs successfully aligned to the *T. truncatus* genome. The final data set therefore included 38 individuals (17 *cases* [neonates and calves, *n* = 9; juveniles, *n* = 6; adults, *n* = 2] and 21 *controls *[juveniles, *n* = 12; adults, *n* = 9]) and 35,493 SNPs that passed quality controls. The remaining missing data averaged only 0.17% per individual (*cases* = 0.10%; *controls* = 0.24%).

### 3.2 Levels of relatedness and inbreeding

Relatedness and inbreeding estimates were based on an unlinked data set (18,285 SNPs). Mean relatedness of pairs of individuals within and between groups were not significantly different (*cases* = −0.0206 ± *SD* 0.05; *controls* = −0.0214 ± *SD* 0.08; *case*–*control* (c–c) = −0.0284 ± *SD* 0.05; all *p* > 0.05; Supporting Information Figure S1), nor was the mean inbreeding coefficient between groups (*cases* = −0.0799 ± *SD* 0.15; *controls* = −0.0853 ± *SD* 0.16, *p* > 0.05; Supporting Information Figure S1).

### 3.3 Genome‐wide association and Random Forest analyses

**Figure 2:**
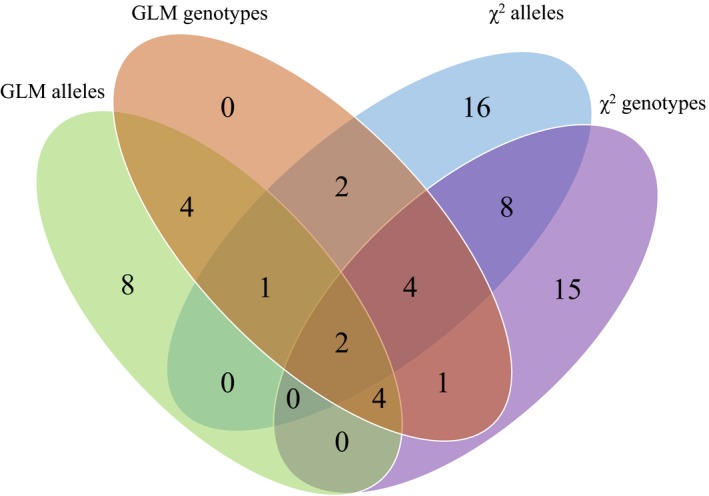
Venn diagram of the number of single nucleotide polymorphisms detected as highly significant in Indo‐Pacific bottlenose dolphins (*Tursiops aduncus*) from the St. Vincent Gulf bioregion, South Australia, used for a genome‐wide association study of cetacean morbillivirus susceptibility and resistance: chi‐square (*χ* ^2^) of allele and genotype frequencies, and generalized linear model (GLM) of allele and genotype frequencies

Of the 35,493 SNPs that successfully aligned to the *T. truncatus* genome, association tests to identify SNPs potentially associated with CeMV resistance or susceptibility resulted in between 12 and 34 highly significant SNPs (*p* < 0.001) depending on the association analysis: chi‐square test or GLM, and allele or genotype frequency (Supporting Information Table S5). Overlap of 26 significant SNPs between association analyses was observed (Figure 2), with a total of 65 significant individual SNPs for all analyses. Subsequently, these SNP subsets were selected for RF analyses (Supporting Information Table S5).

**Figure 3:**
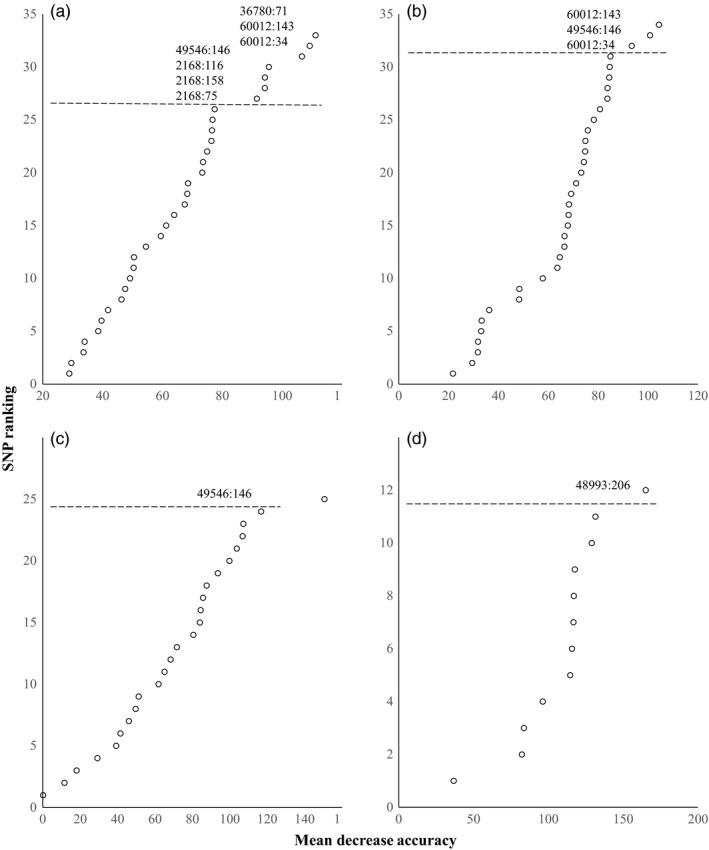
Importance value distributions of the highly significant single nucleotide polymorphism (SNP) subsets in Indo‐Pacific bottlenose dolphins (*Tursiops aduncus*) from the St. Vincent Gulf bioregion, South Australia, used for a genome‐wide association study of cetacean morbillivirus susceptibility and resistance: (a) chi‐square, alleles; (b) chi‐square, genotypes; (c) generalized linear model (GLM), alleles; and (d) GLM, genotypes. The upper elbow of the distribution curve is represented by the horizontal dashed line. SNP ranking: highest number = greater importance

**Figure 4:**
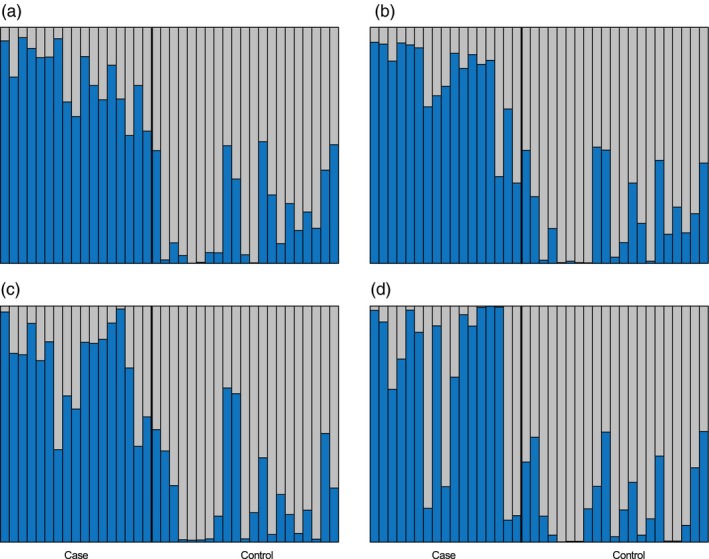
Random Forest classification vote distributions of each Indo‐Pacific bottlenose dolphin (*Tursiops aduncus*) from the St. Vincent Gulf bioregion, South Australia, using the highly significant SNP subsets from a genome‐wide association study of susceptibility and resistance to cetacean morbillivirus: (a) chi‐square, alleles; (b) chi‐square, genotypes; (c) generalized linear model (GLM), alleles; and (d) GLM, genotypes

**Figure 5:**
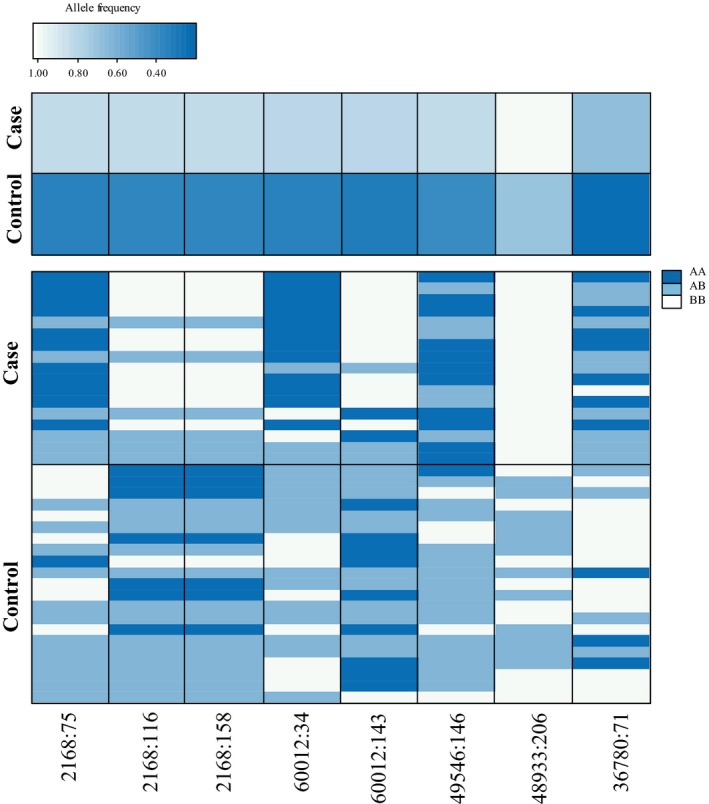
Heatmap of allele and genotype frequencies of the eight candidate SNPs associated with resistance and susceptibility of cetacean morbillivirus in *case* (nonsurvivors) and *control* (putative survivors) Indo‐Pacific bottlenose dolphins (*Tursiops aduncus*) from the St. Vincent Gulf bioregion, South Australia

The RF algorithm reported between 2.63% and 5.41% OOB error rate for distinguishing *case* and *control* individuals, depending on the SNP subset (Supporting Information Table S3). This means that between 94.59% and 97.37% of individuals were correctly classified and that the ranking of variables was generally very reliable for SNP importance selection (Supporting Information Table S3). Using the importance value distributions of the RF analyses, eight SNPs were identified as important markers for classification (Figure 3). Seven SNPs were identified with the SNP subset of the chi‐square test of allele frequencies. Three of the same SNPs were also represented in the chi‐square test of genotypes, and one of the same SNPs in the GLM of alleles, when controlling for age class. In addition, one new SNP was identified with the subset of the GLM of genotype frequencies, when controlling for age class (Figure 3). Visualization of classification votes for the eight candidate SNPs distinguished *case* from *control* individuals well (Figure 4), with permutations resulting in all eight candidates being significantly important for classification (*p* < 0.05; Supporting Information Table S6). Further, the allele and genotype frequencies distinctly differed between *case* and *control* individuals for the eight candidate SNPs (Figure 5), further validating these SNPs as candidates for resistance and/or susceptibility to CeMV.

The eight candidate SNPs placed within five contigs (#2168, #60012, #49546, #36780 and #48933) were successfully aligned to both the *T. truncatus* and *O. orca* genomes, generally with higher alignment scores within the *T. truncatus* genome (Table 2). Four contigs were found within four known genes (*MAPK8*, *INADL*, *ANK3* and *ACOX3*) and one in close proximity to a known gene (*FBXW11*); with their functions putatively associated with stress, pain and immune responses (Table 2). All candidate SNPs within the known genes, however, aligned to intronic regions of these genes (Table 2).

**Table 2:** Summary information of the five candidate genes in the *Tursiops truncatus* (Tt) and *Orcinus orca* (Oo) genomes to which the eight candidate SNPs associated with CeMV susceptibility and resistance in Indo‐Pacific bottlenose dolphins (*Tursiops aduncus*) from the St. Vincent Gulf bioregion, South Australia, were found within or in close proximity

Contig	Accession Tt/Oo	Distance (bp) and genomic region	Predicted gene	% Similarity Tt/Oo	*e*‐value Tt/Oo	Putative function
2168	NW_017843700.1/NW_004438562.1	Within intron	*INADL*	100/100	6e‐96/1e‐89	Formation of tight junctions, regulates expression of *ASIC3* in sensory neurons, epithelial polarity
60012	NW_017842512.1/NW_004438737.1	Within intron	*MAPK8*	99/99	1e‐74/1e‐74	Activates stress related proteins, T‐cell differentiation
49546	NW_017843227.1/NW_004438532.1	Within intron	*ANK3*	97/97	3e‐97/1e‐95	Cell motility, activation and maintenance of specialized membrane domains
36780	NW_017842391.1/NW_004438462.1	12,557/14,860 from intron	*FBXW11*	99/97	4e‐85/9e‐77	Involved in stress‐activated *MAPK* cascade and the T‐cell receptor‐signalling pathway
48933	NW_017844396.1/NW_004438425.1	Within intron	*ACOX3*	98/99	7e‐54/2e‐55	Catalysation of beta‐oxidation for the metabolism of fatty‐acids

Note

Gene functions were derived from UniprotKB (The UniProt Consortium, 2017).

## 4 DISCUSSION

### 4.1 Evidence for CeMV resistance and susceptibility

Using high‐throughput ddRAD sequencing, we successfully generated a genome‐wide data set of 35,493 SNPs for a population of Indo‐Pacific bottlenose dolphins inhabiting the St. Vincent Gulf bioregion, South Australia. We used this resource for a genome‐wide association study of resistance and susceptibility to CeMV from the largest CeMV‐related UME reported to date in the Southern Hemisphere. We found highly significant differences in allele and genotype frequencies between *case* and *control* samples, and RF analysis identified eight SNPs putatively associated with resistance and susceptibility to CeMV in this population. While infectious diseases have long been recognized as powerful selective agents, there have been few studies on the genetic basis of host immune responses in nonmodel species. Here, we uncovered host genetic variants and genes that are likely involved in susceptibility and resistance to a highly contagious and deadly disease in cetaceans. CeMV is currently of particular concern given the rapid expansion in its geographic distribution and number of host species, the discovery of new viral strains and the possibility of vertical transmission (Di Guardo & Mazzariol, 2016; Van Bressem et al., 2014). By identifying genes that are likely involved in virus–host interactions in CeMV infections, our study contributes to a better understanding of the disease biology and host genetic factors involved in susceptibility and resistance. It also enables the development of an applied evolutionary framework to monitor morbillivirus resistance in cetaceans.

### 4.2 Potential influence of relatedness and inbreeding

The association analyses conducted in our study required that *case* and *control* individuals were in general no more related to each other within than between groups (Flint & Eskin, 2012). This was important to avoid biased results due to the presence of genetic structure in the data set. We found no significant difference in the average relatedness within and between *case* and *control* dolphins. This suggests that genetic structure was unlikely to explain the highly significant differences in allele and genotype frequencies observed between *case* and *control *groups at several SNPs.

Inbreeding can lead to an increase in susceptibility to disease (Reid et al., 2007; Spielman, Brook, Briscoe, & Frankham, 2004), making individuals and populations with low diversity more prone to suffering from severe pathogen outbreaks. This was observed during a morbillivirus outbreak of striped dolphins (*Stenella coeruleoalba*) in the Mediterranean between 1990 and 1992. During this outbreak, all stranded animals showed elevated levels of inbreeding, with the dolphins stranding earlier in the outbreak significantly more inbred than those that stranded later in the season (Valsecchi, Amos, Raga, Podestà, & Sherwin, 2004). We found, however, no significant difference in the average inbreeding coefficient between *case* and *control* individuals in our study population, suggesting that the odds of a dolphin succumbing to CeMV during the UME was not associated to its genome‐wide levels of inbreeding.

### 4.3 Annotation and function of candidate genes

Annotation of the five contigs containing the eight SNPs putatively associated with CeMV identified five candidate genes: *MAPK8*, *INADL*, *ANK3*, *FBXW11* and *ACOX3*. These candidate genes are known to be involved with stress, pain and immune responses, which synergistically may have influenced the odds of a dolphin succumbing to CeMV during the UME.

#### 4.3.1 Stress responses

It is well known that stress can lead to a dysregulation of the immune system through its interactions with the central nervous system and the endocrine system (Padgett & Glaser, 2003). Stress induced by environmental changes in particular has been linked to the suppression of immune responses to disease in marine mammals (Wilson et al., 1999). The initial months of the UME and CeMV outbreak in SA were accompanied by abnormally high sea surface temperatures (+3–5°C), and several fish mortality events predominantly on the same side of SVG as most of the dolphin deaths (Kemper et al., 2016). These factors could have led to a more stressful environment to the dolphins, perhaps with less prey available, which has been previously suggested to exacerbate the severity of CeMV outbreaks (Aguilar & Borrell, 1993; Van Bressem et al., 2009). In another marine mammal, atypically high sea surface temperatures in the north‐eastern Pacific have not only negatively impacted upon the body condition of California sea lion pups (*Zalophus californianus*), but also affected their immunocompetence, leading to lower levels of immunoglobulins and reduced capacity to mount an immune response (Banuet‐Martinez et al., 2017).

The candidate gene *MAPK8* is of particular interest due to its involvement in activating stress‐related proteins in response to physiological stress, such as elevated sea surface temperatures (Yu et al., 2004). *MAPKs *are a key group of protein kinases that are highly conserved in eukaryotic cells and function to coordinate responses to stimuli including environmental stressors (Kyriakis & Avruch, 2012). Another of the identified candidate genes, *FBXW11*, which belongs to the *F*‐box protein family, is also involved in the stress‐activated *MAPK* cascade (Liu et al., 2014; Miguel‐Rojas & Hera, 2016). Both, therefore, could have played a pivotal role in responses to the initial environmental stress. In the face of a changing environment, which include extreme weather events and climate anomalies (Luber & McGeehin, 2008), variation at these genes may be very important in the regulation of immune responses.

In the face of stress, vertebrates have evolved the “fight or flight” response, increasing the energy demands of the immune system (Maier, 2003). The candidate gene *ACOX3* is known to be involved in the production of energy, by catalysing the beta‐oxidation of fatty acids to produce energy, CO_2_ and H_2_O (Vanhooren, Marynen, Mannaerts, & Van Veldhoven, 1997). In humans, *ACOX3* expression is generally low in most organs, leading to the suggestion that expression may vary during different developmental stages, or may only be expressed in specialized tissues (Vanhooren et al., 1997). Since the majority of dolphins that died in the South Australian outbreak were young, it is possible that there were also differences in gene expression between age classes which may have led to greater susceptibility of young individuals to CeMV. Variation within this gene has also been suggested to influence the outcome of patients suffering from chronic lymphocytic leukaemia (Wade et al., 2011) and so may influence the risk of cetaceans succumbing to CeMV pathologies related to the lymphoid system.

#### 4.3.2 Pain responses

Pain has been shown to be a powerful stressor in humans and other mammals, with pain‐induced stress resulting in neuroendocrine activation that can induce immune suppression (Page & Ben‐Eliyahu, 1997; Vines, Gupta, Whiteside, Dostal‐Johnson, & Hummler‐Davis, 2003). The candidate gene *INADL* is involved in the expression of a gene proposed to mediate pain, the formation of tight junctions that act as a physical barrier, and it is crucial for cell arrangement, aggregation and epithelial polarity (Roh et al., 2002; Shin, Straight, & Margolis, 2005). In humans, the proteins involved are expressed exclusively in the brain and kidney, regulating the expression or function of *ASIC3* in sensory neurons, a gene proposed to mediate pain induced by acidosis (excessively acidic conditions of bodily fluids or tissues; de Weille, Bassilana, Lazdunski, & Waldmann, 1998). Acidosis is generally associated with poor lung functioning (Bruno & Valenti, 2012), commonly observed in cases of morbillivirus infection; for example, in the SA UME, 97% of the dolphins that tested positive to CeMV suffered from bronchopneumonia (Kemper et al., 2016).

#### 4.3.3 Immune responses

Constantly threatened by pathogenic microorganisms, mammals have evolved an immune system that protects individuals from foreign antigens, and combats symptoms of infection (Herbert & Cohen, 1993). Generally separated into innate and adaptive immune systems, each comprise of both humoural and cellular components, with cellular immune responses mounted against intracellular pathogens such as viruses (Desforges et al., 2016). Cells involved in innate immunity are all‐purpose cells, such as granulocytes, that can quickly attack a number of different pathogens, while those involved in adaptive immunity, such as T‐helper cells, are characterized by a slower response and greater specificity (Segerstrom & Miller, 2004). In cetaceans, mitogen‐activated T‐cell proliferation has been recognized as an important cellular immune response (Beineke, Siebert, Wohlsein, & Baumgärtner, 2010), with reduced T‐cell counts suspected to negatively affect the health status of individuals. *MAPK8* and *FBXW11 *are both part of the T‐cell receptor‐signalling pathway that is involved in the development and differentiation of T cells into Thy‐1 cells (T‐helper cells); cells that help suppress or regulate immune responses (Haeryfar & Hoskin, 2004). In humans and mice, Thy‐1 antibody expression is low during post‐birth development, increasing after maturation (Rege & Hagood, 2016). In humans, MV kills mainly young children who tend to die from complications associated with the disease (Fu et al., 2010). Likewise, the majority of dolphins that succumbed to CeMV during the SA UME were neonates, calves and juveniles. It is therefore possible that expression of Thy‐1 cells was lower for younger dolphins, especially in those with the *case* allele for *MAPK8* and *FBXW11*.

The *MAPK8* gene also has a role in the negative regulation of cell death by apoptotic processes (Haeryfar & Hoskin, 2004). In humans, pro‐inflammatory cytokines, which are released by (macrophages) cells of the innate immune system, and that increase inflammation and worsen symptoms of disease can activate these proteins to promote healing (Kersting et al., 2013). Similarly, *FBXW11 *is a target of the HIV‐1 VPU protein, that can deplete *BST2* (protein coding gene involved in defence responses to viruses) from cells to suppress its antiviral action (Mangeat et al., 2009). Variation within these genes may have influenced an individuals’ ability to fight against or suppress CeMV action to overcome its related pathologies.

Another gene disclosed in our study was *ANK3*, which is part of the ankyrins family and links the integral part of membrane proteins to the cytoskeleton, and is important for cell motility, activation and maintenance of specialized membrane domains (Shirahata et al., 2006). ANK3 can also prevent syncytia (Lang, Wickenden, Wynne, & Lucy, 1984), which can form due to viral fusion proteins, including in the family Paramyxoviridae (Watanabe et al., 2015). In the SA UME, large syncytia, particularly in the lungs, were present in 53% of CeMV‐related dolphin mortalities (Kemper et al., 2016). Therefore, variation within this gene, and potential differences in expression levels, may have provided a protective effect to some individuals, making them more resistant to the virus.

It is also possible that due to the analytical approach employed, some of the candidate SNPs identified may be a false positive (Type I error). For this reason, we have reported these SNPs here as *putatively associated* with CeMV resistance and susceptibility and suggest that when samples from a larger CeMV outbreak are available, further research should seek to confirm their association with CeMV immunity.

### 4.4 Limitations

In this study, we used a GWAS to compare *cases* and *controls* of the 2013 CeMV outbreak in South Australia. While the cases were confirmed to have died from CeMV infection and related pathologies (Kemper et al., 2016) and are true representatives of nonsurvivors of the outbreak, we assume that the *control* samples were exposed to and therefore represent survivors of the CeMV outbreak. This assumption is supported by the population characteristics observed from long‐term studies of this bottlenose dolphin population. That is, the population is relatively small in size and exhibits high social connectivity and low genetic diversity (Pratt et al., 2018; Zanardo et al., 2016). Coupled with the characteristics of CeMV (high rate of transmission, and mode of transmission enhanced by the social nature of dolphins), we believe that the *controls *selected would have been exposed to the virus and indeed represent survivors of the outbreak.

Large sample sizes are generally very difficult to achieve when working with wildlife diseases, particularly in marine species that exhibit nonsedentary and often pelagic characteristics (Gulland & Hall, 2007). In these circumstances, studies rely primarily on stranding events. Due to the size of the SVG CeMV outbreak (29 *cases* available), this study was limited in the number of samples, and using the available samples through a ddRAD approach, it covered only approximately 1% of the dolphin genome. It is likely that other genes are also involved in resistance and susceptibility to CeMV but were not detected, either due to low power (false negatives, Type II error) or coverage (not sampled). Given the highly polygenic nature of many infectious diseases, including the related MV (Haralambieva et al., 2015), it is expected that other immune genes would also be involved with CeMV. For instance, the MHC family has been among some of the most studied immune response genes because of their high variability and importance in antigen recognition, and variation has found to be associated with resistance to HAB’s in bottlenose dolphins (Cammen, Schultz, et al., 2015; Cammen, Wilcox, Rosel, Wells, & Read, 2015). There are also at least 16 immune response genes (listed in the introduction) that are known to encode key proteins involved in host cellular interactions with morbilliviruses (Haralambieva et al., 2015; McCarthy et al., 2011; Stejskalova et al., 2017), which were either partially sequenced but not observed as being significantly associated with CeMV resistance or susceptibility in our study, or were not sequenced at all (data not shown). While a targeted candidate gene approach allows an in‐depth analysis of known immune genes with functional importance, here we have uncovered and provided evidence for the importance of stress, pain and immune‐related genes for CeMV resistance and susceptibility using a genome‐wide association study of a dolphin UME and CeMV outbreak. For a comprehensive understanding of the host genetics of CeMV infection, ideally future studies would expand analyses to the whole genome, utilize larger sample sizes and of several populations, and of outbreaks involving different cetacean species and CeMV strains.

### 4.5 Vaccination against CeMV

As the climate changes, and extreme weather events become more frequent, marine populations face growing levels of stress that may negatively impact on individual and population health. For example, periods of thermal stress have been implicated in CeMV, PDV and CDV outbreaks (Aguilar & Borrell, 1993; Burge et al., 2014; Kemper et al., 2016; Kuiken et al., 2006; Lavigne & Schmitz, 1990), while climate change could alter the incidence and prevalence of disease outbreaks and the severity of infection (Burge et al., 2014). With this in mind, large CeMV mortality events appear to be increasing in incidence, geographic distribution and number of host species, particularly since the late 1980s, highlighting the need for strategies to minimize these outbreaks and mortalities, including the potential use of immunizations. In humans, vaccines that induce immunity against MV, which is a live attenuating virus including measles, mumps and rubella, have been promising and effective (Marin et al., 2006). In cetaceans, vaccines against CeMV, targeting the fusion (F) and hemagglutinin (H) genes, have been trialled and shown partial success in U.S. Naval trained dolphins (*T. truncatus*; Vaughan et al., 2007). In wild cetaceans, challenges for vaccine implementation would include time and costs associated with large‐scale administration and health risks associated with attenuated live viral vaccines (Duignan et al., 2014). In pinnipeds, vaccination tests against PDV in captive Hawaiian monk seals (*Monachus schauinslandi*) have shown success in vaccine selection, safety and efficacy, with strategies now under investigation for the vaccination of free‐ranging individuals (Duignan et al., 2014; Quinley et al., 2013). In this study, we have uncovered five candidate genes likely involved in responses to stress and pain and immune responses to CeMV, enhancing our understanding of resistance and susceptibility to this disease, and potentially aiding further advances of vaccines against morbilliviruses.

## 5 CONCLUSIONS

To the best of our knowledge, this is the first cetacean study to provide empirical evidence about genetic variants associated with resistance and susceptibility to CeMV. In summary, we revealed five candidate genes putatively associated with resistance and susceptibility to CeMV using high‐throughput ddRAD sequencing. *MAPK8* and *FBXW11* are both part of the *MAPK* cascade and seem to be involved with resistance to stress and in immune responses, while *INADL* is involved in pain responses, *ACOX3* in energy production, and *ANK3* with cellular responses. These findings provide the first insights into the genetic basis of host defence to this highly contagious disease and are useful for the future development of biomarkers for CeMV resistance and susceptibility, and in the potential advancement of immunizations against morbilliviruses. Future studies should aim to screen other CeMV affected populations and species for signals of selection within these candidate genes and investigate further stress, pain and immune related genes, which may be associated with this highly virulent and fatal disease.

## DATA AVAILABILITY

Reference sequences and SNP genotypes are available at the Dryad Digital Repository: https://doi.org/10.5061/dryad.tk8774f.

## CONFLICT OF INTEREST

None declared.

## AUTHOR CONTRIBUTIONS

The study was designed by LM, in conjunction with LB and KB. Biopsy samples of live dolphins were collected by LM and NZ, while tissue samples from diseased dolphins and information about these were provided by CK and IT. Laboratory work was primarily conducted by KB, with guidance and assistance from CA. KB and JSC performed the bioinformatics, while data analysis was primarily carried out by KB and LM, with guidance from JSC, CA and LB. KB and LM wrote the manuscript, while LB, JSC, CA, CK and IT critically revised it.
