# Supplementary material for: Genome‐wide association study of an unusual dolphin mortality event reveals candidate genes for susceptibility and resistance to cetacean morbillivirus
Source: Evol Appl. 2018 Dec 26;12(4):718–32. doi: 10.1111/eva.12747 (PMC6439501; doi:10.1111/eva.12747)
Supplement: Supplementary file 1 [file EVA-12-718-s001.docx]

**Supplementary Information**

**Genome-wide association study of an unusual dolphin mortality event reveals candidate genes for susceptibility and resistance to cetacean morbillivirus**

**
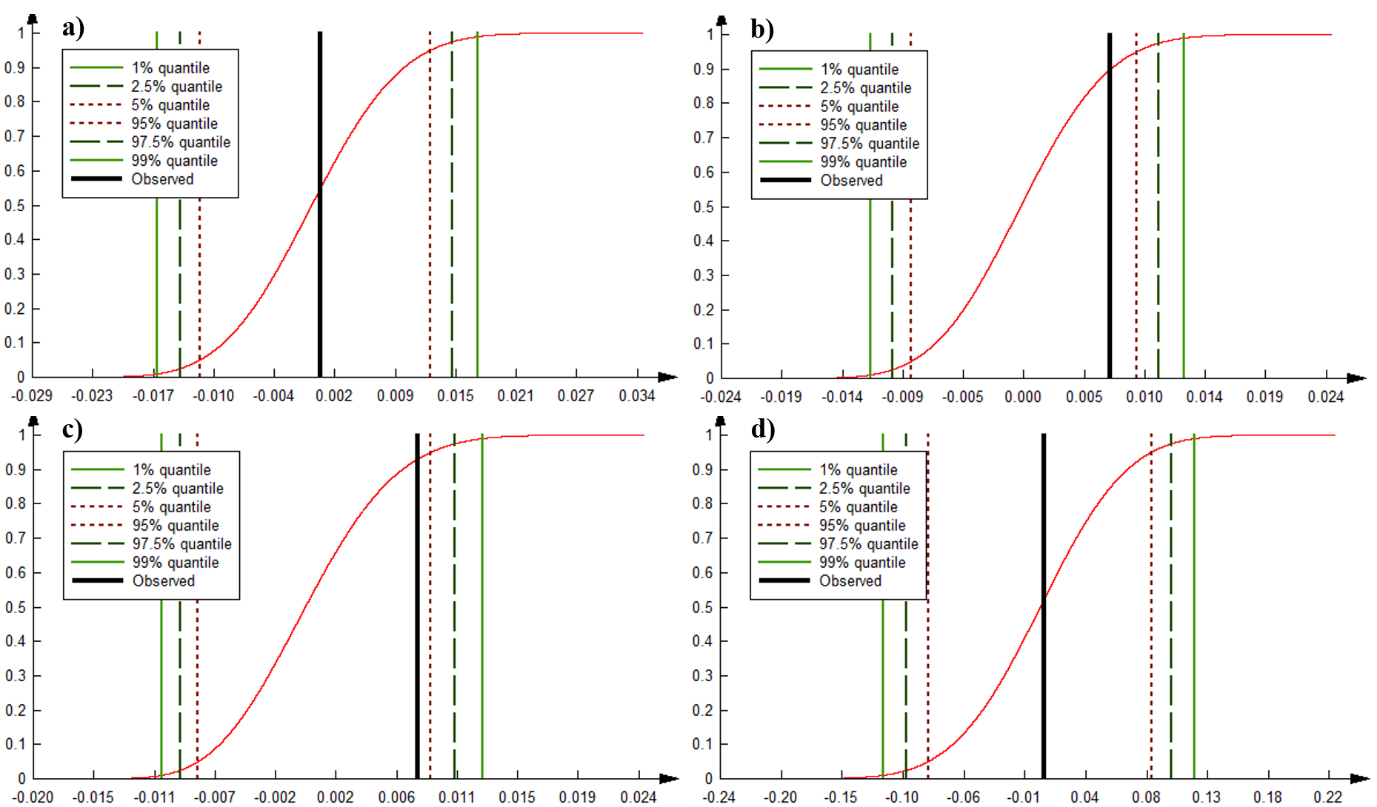
**

**Figure S1**: Cumulative frequency distributions of relatedness and inbreeding of *case* (CA) and *control* (CO) Indo-Pacific bottlenose dolphins (*Tursiops aduncus*) from the St. Vincent Gulf bioregion, South Australia, used for a genome-wide association study of cetacean morbillivirus susceptibility and resistance. Ritland estimator of relatedness between: a) CA vs CO; b) CO vs CA-CO; c) CA vs CO-CA; and Ritland estimator of inbreeding between: d) CA vs CO.

**Table S1.** Sex and age data of selected *case* and *control* Indo-Pacific bottlenose dolphins (*Tursiops aduncus*) of an unusual dolphin mortality event and cetacean morbillivirus outbreak in the St. Vincent Gulf bioregion, South Australia, used for double digest Restriction Associated DNA sequencing.

| **Age class** | **Young** | | | **Adult** | | | **Total** |
| --- | --- | --- | --- | --- | --- | --- | --- |
| **Sex** | **M** | **F** | **Total young** | **M** | **F** | **Total adult** |  |
| *Case* | 8 | 7 | 15 | 2 | 0 | 2 | 17 |
| *Control* | 7 | 6 | 13 | 6 | 3 | 9 | 22 |
| **Total** | 15 | 13 | 28 | 8 | 3 | 11 | 39 |
|  |  |  |  |  |  |  |  |

**Table S2.** Average depth per contig per individual for 38 *case* and *control* Indo-Pacific bottlenose dolphins (*Tursiops aduncus*) from an unusual dolphin mortality event and cetacean morbillivirus outbreak in the St. Vincent Gulf bioregion, South Australia.

| **Treatment** | **Individual/ABTC** | **# SNPs** | **Depth** |
| --- | --- | --- | --- |
| CA | 139232 | 35445 | 29.4072 |
| CA | 139236 | 35487 | 52.4527 |
| CA | 139239 | 35482 | 47.1497 |
| CA | 139240 | 35477 | 24.9786 |
| CA | 139242 | 35484 | 26.2113 |
| CA | 139243 | 35491 | 61.3529 |
| CA | 139250 | 35460 | 17.9415 |
| CA | 139251 | 35483 | 64.676 |
| CA | 139327 | 35465 | 80.6214 |
| CA | 139329 | 35306 | 24.8251 |
| CA | 139330 | 35466 | 25.5006 |
| CA | 139331 | 35455 | 28.538 |
| CA | 139334 | 35481 | 38.5512 |
| CA | 139339 | 35407 | 32.8593 |
| CA | 139340 | 35425 | 19.9693 |
| CA | 139341 | 35488 | 35.6825 |
| CA | 139343 | 35437 | 33.3177 |
| CO | AD13 | 35485 | 31.7517 |
| CO | AD28 | 35485 | 49.6793 |
| CO | AD29 | 35460 | 44.4648 |
| CO | AD34 | 35403 | 13.9278 |
| CO | AD35 | 35489 | 36.1345 |
| CO | AD39 | 35455 | 20.3686 |
| CO | AD44 | 35448 | 27.167 |
| CO | AD47 | 35483 | 30.8132 |
| CO | AD49 | 35484 | 29.4229 |
| CO | AD59 | 35123 | 11.4328 |
| CO | AD70 | 35196 | 18.4035 |
| CO | AD76 | 35303 | 14.5807 |
| CO | AD77 | 35457 | 27.7021 |
| CO | AD96 | 35489 | 35.583 |
| CO | AD98 | 35483 | 39.618 |
| CO | AD99 | 35367 | 15.5812 |
| CO | CJ143 | 35415 | 20.6936 |
| CO | CJ144 | 35445 | 33.5863 |
| CO | PW68 | 35230 | 18.383 |
| CO | SB108 | 35401 | 23.8366 |
| CO | SB109 | 35455 | 39.3844 |

^Individual AD12 was removed during filtering due to high levels of missing data. ABTC (Australian Biological Tissue Collection) numbers are provided for SA museum samples only.

**Table S3.** Number of randomly chosen SNPs considered in each tree split (mtry) and out-of-bag (OOB) error rates for distinguishing *case* and *control* Indo-Pacific bottlenose dolphins (*Tursiops aduncus*) from the St. Vincent Gulf bioregion, South Australia, for the Random Forest analyses of cetacean morbillivirus susceptibility and resistance.

| **Analysis** | **Data** | **SNPs** | **OOB (%)** | **mtry** |
| --- | --- | --- | --- | --- |
| **Chi-square** | Alleles | 33 | 2.3 | 6 |
|  | Genotypes | 34 | 5.41 | 2 |
| **GLM** | Alleles | 25 | 2.63 | 6 |
|  | Genotypes | 12 | 2.63 | 4 |

**Table S4.** Illumina HiSeq2500 sequencing statistics for 41 *case* and *control* Indo-Pacific bottlenose dolphin (*Tursiops aduncus*) from an unusual dolphin mortality event and cetacean morbillivirus outbreak in the St. Vincent Gulf bioregion, South Australia.

| **Read statistics** | **# of reads** |
| --- | --- |
| Raw reads | 525,785,802 |
| Filtered reads | 278,147,988 |
| Average reads per sample | 6,784,097 |
| Minimum reads per sample | 2,445,976 |
| Maximum reads per sample | 17,741,388 |

**Table S5:** Summary of results from testing for associations between genetic variation of 35,493 single nucleotide polymorphisms (SNPs) and cetacean morbillivirus susceptibility and resistance in Indo-Pacific bottlenose dolphins (*Tursiops aduncus*) from the St. Vincent Gulf bioregion, South Australia.

| **Analysis** | **Data** | **SNPs**  **(P <0.001)** | **Contig (SNPs)** |
| --- | --- | --- | --- |
| Chi-square | Alleles | 33 | 106275(149), 19012(104,124), 2168(24,75,116,158), 25181(120,255), 29510(27), 31019(77), 36780(71), 37677(100), 45573(58), 48933(206), 49546(146), 50004(79), 56617(110,204), 60012(34,143), 61732(145), 61844(24,51), 61966(154), 64000(70), 70993(202), 73642(98), 76528(4), 82060(63,89,187), 87040(65) |
|  | Genotypes | 34 | 19012(104,124), 19897(28,65,160,197), 2168(24,75,116,158), 26564(173,178,181,186), 30286(137), 35211(179), 36780(71), 42634(226), 45573(58), 48933(206), 49546(146), 52279(190,205), 60012(34,143), 61732(145), 67940(52), 70993(202), 73168(44,48), 76208(125), 77170(213), 78873(82,185) |
|  |  |  |  |
| Generalised Linear Model | Alleles | 25 | 19897(28,65,160,197), 2168(75,116,158), 29510(27), 31019(77), 35719(15), 48676(213,219), 48993(206), 49546(146), 52591(214), 55029(144), 55674(243), 58823(233), 60417(97), 70993(202), 79489(96), 85771(25), 87040(65), 9537(137,157) |
|  | Genotypes | 12 | 19897(28,65,160,197), 31019(77), 42634(226), 48933(206), 52591(214), 60417(97), 70993(202), 85771(25), 9537(137) |

**Table S6.** *P*-values (*P*-val) of importance value distributions and classification votes for the eight candidate SNPs identified in the Random Forest analyses as associated with cetacean morbillivirus susceptibility and resistance in Indo-Pacific bottlenose dolphins (*Tursiops aduncus*) from the St. Vincent Gulf bioregion, South Australia.

| **Analysis** | **Data** | **Contig(SNP)** | **Importance values** | **Case**  **(*P*-val)** | **Control**  **(*P*-val)** |
| --- | --- | --- | --- | --- | --- |
| Chi-square | Alleles | 60012(143) | 0.0099 | 0.0099 | 0.0099 |
|  |  | 36780(71) | 0.0099 | 0.0099 | 0.0495 |
|  |  | 60012(34) | 0.0099 | 0.0099 | 0.0099 |
|  |  | 49546(146) | 0.0297 | 0.0297 | 0.0198 |
|  |  | 2168(116) | 0.0099 | 0.0099 | 0.0099 |
|  |  | 2168(158) | 0.0099 | 0.0099 | 0.0099 |
|  |  | 2168(75) | 0.0099 | 0.0099 | 0.0198 |
|  | Genotypes | 60012(143) | 0.0099 | 0.0099 | 0.0198 |
|  |  | 49546(146) | 0.0099 | 0.0099 | 0.0099 |
|  |  | 60012(34) | 0.0099 | 0.0099 | 0.0297 |
| Generalised Linear Model | Alleles | 49546(146) | 0.0099 | 0.0099 | 0.0099 |
|  | Genotypes | 48933(206) | 0.0198 | 0.0099 | 0.0297 |
